# Translation, cultural adaptation and validation of Patient Health Questionnaire and generalized anxiety disorder among adolescents in Nepal

**DOI:** 10.1186/s13034-024-00763-7

**Published:** 2024-06-19

**Authors:** Nagendra P. Luitel, Damodar Rimal, Georgia Eleftheriou, Kelly Rose-Clarke, Suvash Nayaju, Kamal Gautam, Sagun Ballav Pant, Narmada Devkota, Shruti Rana, Jug Maya Chaudhary, Bhupendra Singh Gurung, Jill Witney Åhs, Liliana Carvajal-Velez, Brandon A. Kohrt

**Affiliations:** 1 Department of Global Public Health, Karolinska Institutet, Stockholm, Sweden; 2 Research Department, Transcultural Psychosocial Organization (TPO) Nepal, Baluwatar, Kathmandu Nepal; 3 Center for Global Mental Health Equity, Department of Psychiatry and Behavioural Health, The George Washington University, Washington, D.C USA; 4 Department of Global Health and Social Medicine, King’s College London, London, UK; 5 Department of Psychiatry & Mental Health, Institute of Medicine, Tribhuvan University, Kathmandu, Nepal; 6 Child and Adolescent Psychiatry Unit, Kanti Children’s Hospital, Kathmandu, Nepal; 7 Division of Data, Analytics, Planning and Monitoring, Data and Analytics Section, UNICEF, New York, New York USA

**Keywords:** Depression and anxiety, Adolescents, Cultural adaptation, Validation, Nepal

## Abstract

**Background:**

Depression and anxiety are significant contributors to the global burden of disease among young people. Accurate data on the prevalence of these conditions are crucial for the equitable distribution of resources for planning and implementing effective programs. This study aimed to culturally adapt and validate data collection tools for measuring depression and anxiety at the population level.

**Methods:**

The study was conducted in Kathmandu, Nepal, a diverse city with multiple ethnicities, languages, and cultures. Ten focus group discussions with 56 participants and 25 cognitive interviews were conducted to inform adaptations of the Patient Health Questionnaire adapted for Adolescents (PHQ-A) and Generalized Anxiety Disorder (GAD-7). To validate the tools, a cross-sectional survey of 413 adolescents (aged 12–19) was conducted in three municipalities of Kathmandu district. Trained clinical psychologists administered the Kiddie Schedule for Affective Disorders and Schizophrenia (K-SADS-DSM 5 version) to survey participants.

**Results:**

A number of cultural adaptations were required, such as changing statements into questions, using a visual scale (glass scale) to maintain uniformity in responses, and including a time frame at the beginning of each item. For younger adolescents aged 12 to 14 years, a PHQ-A cut-off of > = 13 had a sensitivity of 0.93, specificity of 0.80, positive predictive value (PPV) of 0.33, and negative predictive value (NPV) of 0.99. For older adolescents aged 15–19, a cut-off of > = 11 had a sensitivity of 0.89, specificity of 0.70, PPV of 0.32, and NPV of 0.97. For GAD-7, a cut-off of > = 8 had a sensitivity of 0.70 and specificity of 0.67 for younger adolescents and 0.71 for older adolescents, with a PPV of 0.39 and NPV of 0.89. The individual symptom means of both PHQ-A and GAD-7 items showed moderate ability to discriminate between adolescents with and without depression and anxiety.

**Conclusion:**

The PHQ-A and GAD-7 demonstrate fair psychometric properties for screening depression but performed poorly for anxiety, with high rates of false positives. Even when using clinically validated cut-offs, population prevalence rates would be inflated by 2–4 fold with these tools, requiring adjustment when interpreting epidemiological findings.

**Supplementary Information:**

The online version contains supplementary material available at 10.1186/s13034-024-00763-7.

## Introduction

 Adolescence is the peak age for the onset of 50% of mental health conditions (Kessler et al. [16]). Depressive and anxiety disorders make up over 50% of the total estimated mental health burden among adolescents (Institute of Health Metrics (IHME), [12]). Additionally, suicide was the fourth leading cause of death among 15-29-year-olds globally in 2019 (World Health Organization [39]). Negative impacts associated with depression and anxiety disorders include failure to complete secondary school, unemployment, and unplanned pregnancy or parenthood (Cleary et al. [6]). These adverse consequences disproportionately affect the more than 1 billion adolescents living in low- and middle-income countries (LMICs) (Hayes et al. [10]).

Adolescents in Nepal are at high risk of mental health problems due to recent and historical trauma, including two major earthquakes in 2015, civil war from 1996 to 2006 and recurrent natural disasters such as seasonal flash floods and landslides. This compounded by parental migration and socio-economic deprivation. Limited research has been conducted to assess mental health problems among children and adolescents in Nepal. Existing studies show a wide variation in reported rates of depression ranging from 0.4 to 56.5%, (Dhimal et al. [8]; Gautam et al. [9]; Karki et al. [14]; Ojha et al. [28]; Paudel et al. [29]; Risal and Sharma [32]; Sharma et al. [33]; Silwal et al. [34]) and anxiety ranging from 2 to 55.6% (Dangal and Bajracharya [7]; Dhimal et al. [8]; Karki et al. [14]; Ojha et al. [28]; Paudel et al. [29]; Rimal and Pokharel [31]) among children and adolescents. The wide range of reported prevalence rates could be due to the range of tools used, non-random sampling and small sample sizes (Steel et al. [36]). Research on adolescent mental health in LMICs like Nepal is hindered by the lack of validated measurement tools that can be used at the population level. Cultural adaptation and validation of tools are needed to accurately assess the mental health burden (Kohrt and Kaiser [19]) and inform the development and implementation of mental health policies and services (Hayes et al. [10]). Similarly, studies have pointed out that early identification and treatment of these disorders are the key factors for improved prognosis (McGorry and Mei [24]).

This study aims to address gaps in measuring depression and anxiety among adolescents in Nepal at the population level. In this paper, we describe a systematic process to translate, culturally adapt, and validate the Patient Health Questionnaire adapted for adolescents (PHQ-A) and Generalized Anxiety Disorder (GAD-7) for adolescents aged 12 to 19. This process follows the protocol developed as part of UNICEF’s initiative to measure adolescent and young people’s mental health at the population level (MMAPP) (Carvajal-Velez et al. [4]). The cultural adaptation and clinical validation ensure the accuracy of assessment tools to improve the detection of adolescents with mental health conditions. This data can be used in future health-promotion efforts and healthcare resource allocation. Ultimately, the data generated by these tools may guide the appropriate allocation of resources to address adolescent mental health conditions and inform decisions about prioritizing investment in interventions to prevent or alleviate the impact of mental health conditions among adolescents.

## Methods

### Setting

The study was conducted in Kathmandu district, the capital city of Nepal, which has a total population of approximately two million (National Statistics Office [27]). Nepal is home to more than 125 caste/ethnic groups, many of whom have their own language and culture (Central Bureau of Statistics [5]). There are 123 languages spoken including Nepali, the national language. The national literacy rate is 76.2% (National Statistics Office [27]) with school enrollment at 94.7% in the basic level (grades 1 to 8) and 51.2% in the secondary level (Ministry of Education Science and Technology [25]). Focus group discussions (FGDs) and cognitive interviews were conducted in Nagarjun Municipality while the validation study was conducted in Kathmandu Metropolitan City, Budhanilkantha Municipality and Tokha Municipality of Kathmandu district.

### Participants and sample size

The study included Nepali-speaking adolescents aged 12 to 19, representing diverse castes, ethnicities, languages, and cultures. We aimed to recruit 500 adolescents, divided into two age groups: 12 to 14 and 15 to 19. The study had two phases: Phase 1 involved qualitative research, including FGDs and CIs to translate and adapt the tool, while Phase 2 included survey questionnaires and semi-structured clinical interviews. In Phase-I, 81 adolescents participated in activities to adapt the PHQ-A and GAD-7 for the Nepali context. This included 10 FGDs (56 participants) and 25 cognitive interviews conducted between July and August 2021. FGDs were held separately for boys and girls, as well as younger and older adolescents. In Phase 2, 413 adolescents completed the quantitative survey for the validation component (157 younger adolescents and 256 older adolescents).

**Fig. 1:**
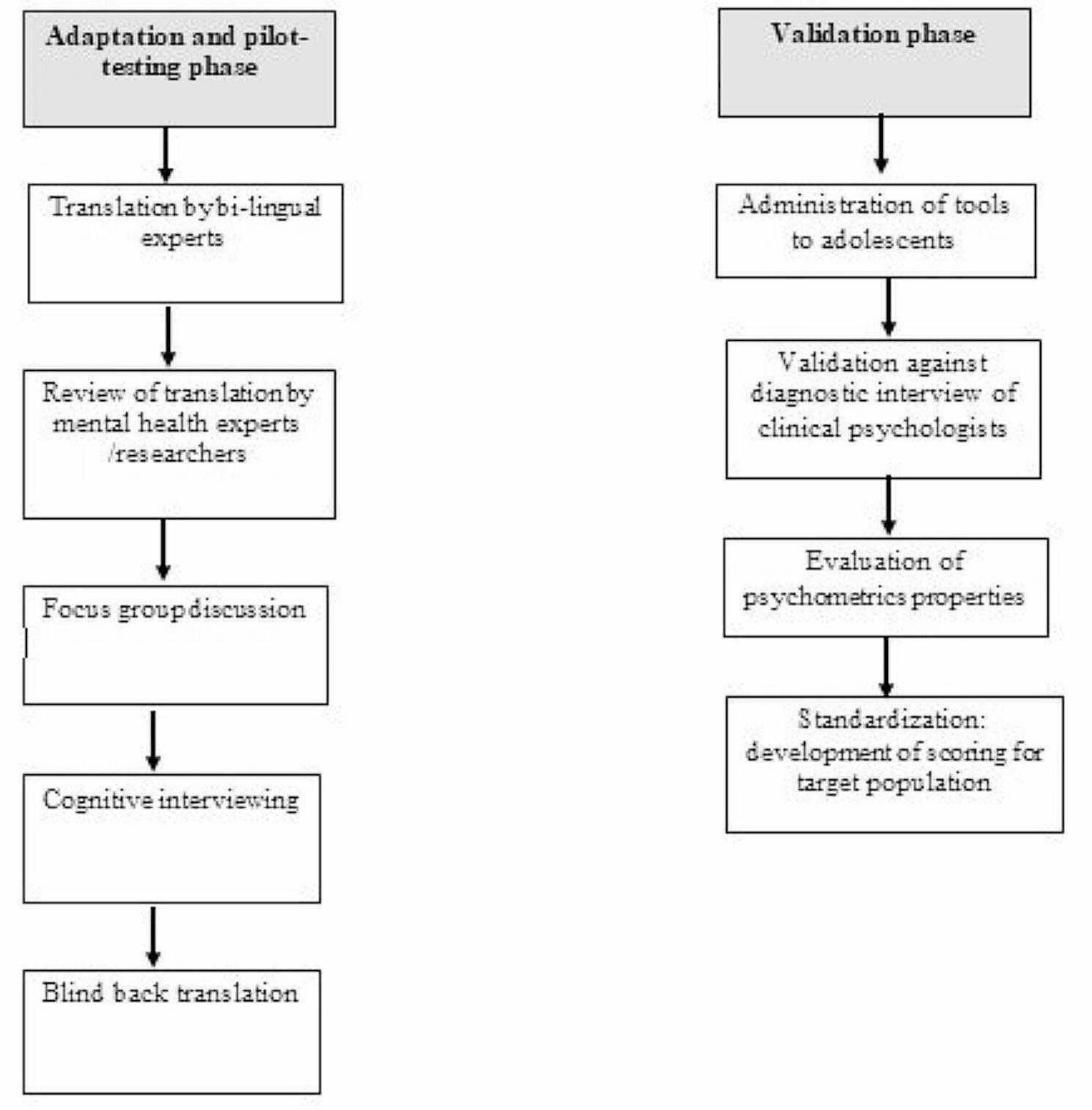
Study process and flow chart

 Figure 1 summarizes the study process and steps followed in both the adaptation and pilot testing, and validation phases.

### Recruitment process

Two trained and experienced researchers oversaw both Phase 1 qualitative (focus group discussions and cognitive interviews) and Phase 2 quantitative survey administration. Research assistants (RAs) received a two-week training that covered both qualitative and quantitative data collection. The training focused on unstructured and structured interviewing, study population, sample size, and recruitment process. It also covered the content of the instruments and interview guides, scoring, referral systems, immediate care, and inclusion/exclusion criteria. Various pre-tests and mock interviews were conducted during the training period to assess the interviewers’ confidence levels.

### Phase-I recruitment

The focus group discussions and cognitive interviews were conducted with school-going adolescents from Nagarjun Municipality, Budhanilkantha Municipality, and Kathmandu Metropolitan City, all located in the Kathmandu Valley. Participants for the FGDs and CIs were selected with the support from school teachers and principals. Purposive sampling was used to ensure representation of different castes/ethnicities and age groups. After receiving the name list from the schools, we distributed consent forms to the adolescents. Those who returned the signed forms were included in the focus group discussions and cognitive interviews. Additionally, we conducted interviews with adolescents receiving services from TPO Nepal.

### Phase II recruitment

We aimed to recruit a diverse sample of participants, some of whom were likely to have anxiety or depression, and others who did not have these conditions. We used proactive case detection strategies, specifically the Community Informant Detection Tool (CIDT) (Jordans et al. [13]) to recruit participants from different public schools in Budhanilkantha and Tokha Municipalities. First, a psychosocial counselor trained school teachers on the CIDT for anxiety and depression, asking them to refer adolescents who met the CIDT threshold for anxiety or depression, as well as those who did not meet any CIDT criteria. When we were unable to recruit enough adolescents through teachers, the psychosocial counselor also trained students on the CIDT for self-referral. To increase the number of adolescents likely to have depression or anxiety, we also recruited some adolescents from the Child & Adolescent Psychiatry (CAP) unit of Kanti Children’s Hospital (KCH), the only dedicated child and mental health service provider in Nepal. Research assistants (RAs) visited each school or KCH, assessed inclusion/exclusion criteria, obtained written informed assent and consent, and conducted interviews in a confidential space, such as a spare room or open ground.

Four clinical psychologists specializing in child and adolescent populations conducted clinical interviews using the Kiddie Schedule for Affective Disorders and Schizophrenia (K-SADS). They received training on identifying depressive and anxiety disorders, interviewing techniques, K-SADS administration, and referrals from an expert psychiatrist. They also established inter-rater reliability through practice assessments with adolescents. Both research assistants (RAs) and clinical psychologists used Android-supported devices to administer the PHQ-A and GAD-7, as well as the K-SADS interview schedules. Of the 413 participants, 236 were first interviewed by RAs, while 177 were first interviewed by the clinical psychologists. The second interviews were conducted within 72 h of the first interview. The clinical psychologists conducting the interviews were blind to the results of the PHQ-A and GAD-7. The entire interview process, including the PHQ-A, GAD-7, and K-SADS, lasted between 1 and 1.15 h. Data collected by RAs and clinical psychologists on Android devices were submitted online daily for quality assurance. Data collection took place between September 1, 2021, and December 21, 2021, and was conducted in Nepali for both Phase-I and Phase-II.

### Phase-I interview guide

The FGD guide included questions to assess the acceptability, relevance, comprehensibility, and completeness of each item in the tool. It also included pictorial and Likert scales (Kohrt et al. [18]) to determine the best structure for eliciting responses. The guide also included questions to check participants’ understanding by asking them to rephrase the questions in their own words. The CI guide included questions to determine how participants interpret the items and response options in the tool. It also included questions to assess the extent to which adolescents associate symptoms with impairment in daily functioning. The guide also allowed participants to modify and change the questions in their own language.

### Phase-II instruments

*Patient health questionnaire adapted for adolescents (PHQ-A)*: PHQ-A is a widely used tool for assessing depressive symptoms in adolescents aged 11 to 17 [30]. Adolescents are asked to score nine common symptoms of depression based on their experience in the past 2 weeks. It has a four-point rating scale that ranges from 0 ‘not at all’ to 3 ‘always. The raw scores on the 9 items should be summed to obtain a total. The total score ranges from 0 to 27, with higher scores indicating greater severity of depression. The PHQ-A is based on the PHQ-9 which was previously shown to be a reliable and valid tool to assess depressive symptoms among adults in Nepal (Kohrt et al. [20]). A cut-off of 10 was recommended to indicate moderate and severe depression.

*Generalized Anxiety Disorder (GAD-7*): GAD-7 is a seven-item tool that is used to screen for generalized anxiety disorder. The GAD-7 is a self-administered questionnaire and each item has a Likert-response format on a 4-point scale (0–3 points). Respondents were asked to consider the previous 2 weeks and to rate symptom frequency as ‘not at all’ (0), ‘several days’ (1), ‘more than half of all days’ (2) or ‘nearly all days’ (3). The total score response ranged from 0 to 21 (Spitzer et al. [35]). GAD-7 has been validated in various settings, and has widely been used to measure anxiety disorders (Mughal et al. [26]).

*Kiddie Schedule for Affective Disorders and Schizophrenia (K-SADS)*: We used the K-SADS as the standard diagnostic measure for depression and anxiety. The K-SADS is a semi-structured interview for children aged 6–18, designed to assess ongoing psychopathology, including mood and anxiety disorders (Kaufman et al. [15]). Modules include a screening section to determine if a more comprehensive section is needed. Symptoms are scored as 2 (subthreshold) or 3 (threshold), and criteria-based algorithms determine the presence of current disorders, in line with the Diagnostic and Statistical Manual of Mental Disorders (DSM-5) (American Psychiatric Association [1]). For this study, clinical psychologists administered the depression module and a selection of anxiety modules (generalized anxiety, social anxiety, panic disorder, and separation anxiety) of the K-SADS to identify major depressive disorder and anxiety disorders. The clinical psychologists were trained and supervised by a bilingual psychiatrist. The K-SADS was previously translated, adapted and used in Nepal (Kohrt et al. [18]).

### Statistical analyses

We manually analyzed Phase 1 qualitative data using the content analysis method. In Phase II, we presented the data descriptively for the full sample and stratified by gender. We used diagnostic categories including depression diagnosis, anxiety diagnosis, any diagnosis, and no diagnosis. To assess the criterion validity of the PHQ-A and GAD-7 against a clinician’s diagnosis (K-SADS), we constructed a receiver operating characteristic (ROC) curve using the PHQ-A and GAD-7 total scores. We calculated the area under the curve (AUC) separately for boys, girls, younger adolescents, and older adolescents, using the K-SADS as the gold standard. Additionally, we calculated psychometric properties for a range of cut-off scores and presented sensitivity, specificity, positive predictive value (PPV), and negative predictive value (NPV). We compared individual items of both the PHQ-A and GAD-7 between adolescents with depression/anxiety and those without any diagnosis using an independent t-test. Statistical analysis was performed using the Statistical Package for Social Science (SPSS) version 28 (IBM Corp [11]).

## Results

### Phase-1 qualitative results

Out of the 81 adolescents who participated in the Phase-I qualitative study, the majority were between the ages of 12 and 15 (58%), currently in grades 6 to 8 (38.3%), and belonged to the Brahmin/Chhetri (45.7%) or Janajati (34.6%) caste/ethnic groups.

### Translation and cultural adaptation of PHQ-A and GAD-7

The translation and cultural adaptation of the PHQ-A and GAD-7 were conducted following UNICEF’s MMAPP protocol (Carvajal-Velez et al. [4]). The PHQ-A was translated by two mental health experts, including a clinical psychologist and a psychiatrist with a research background, who were experienced in translating mental health tools. The GAD-7 was translated by three mental health experts, including two clinical psychologists and one psychiatrist. The translators followed the guidelines recommended for cross-cultural research (van Ommeren et al. [38]).

First, a particular item of the PHQ-A and GAD-7 was translated from English to Nepali, back translated into English, and evaluated based on comprehensibility, acceptability, relevance, and completeness (Kohrt et al. [18]). Second, the translations were reviewed by two mental health researchers and one psychiatrist, who made several adaptations to ensure the translations were acceptable and easily understandable by different groups of adolescents. Third, feedback and inputs from adolescents were obtained through focus group discussions (FGDs) and cognitive interviews (CIs). In the FGDs, participants were asked if the translations were understandable, acceptable, and relevant to adolescents from different caste/ethnic groups, ages, and education levels. If a word or terminology was not acceptable or understandable, participants were asked to provide an alternative translation or terminology. Fourth, after incorporating feedback and suggestions from the FGDs, cognitive interviews were conducted individually with adolescents who were not involved in the FGDs. The results from the FGDs and CIs are summarized separately for the PHQ-A and GAD-7 in Tables 1 and 2, respectively.

**Table 1:** PHQ-A original item, back-translation of Nepali items, and comments from adolescents

Original Items	Back translation of the final Nepali version	Some examples of comments made by participants
Feeling down, depressed, irritable, or hopeless	In the past 2 weeks, how much did you feel down, depressed, irritable or hopeless?	– Most of the adolescents understood this item – Adolescents felt that feeling down (*nirash hune*) and feeling depressed (*udash hune*) have same meaning in Nepali language – Adolescents reported that when they do not get good marks or fail in school examinations they feel down – *We know when someone is sad or depressed by their facial expression. They seem so depressed. People feel sad when they do not get success in what they have attempted, or when they cannot fulfill their family’s wishes* – *If someone is putting hand in her/his head and making a sad face, then we know that he/she is depressed*
Little interest or pleasure in doing things	In the past 2 weeks, how much have you been less interested or had pleasure doing things that you really used to enjoy? (e.g. reading, writing, playing games, hanging out, using mobile, etc.)	– Some adolescents had difficulty in understanding this item – An example (writing, playing games, hanging out, using mobile) has been added to make the term *“things”* clear – Participants reported that adolescents who have gone through bad experiences (doing the things) or have stress/tension might feel little interest in reading, travelling and might only want to stay in home
Trouble falling asleep, or staying asleep or sleeping too much	In the past 2 weeks, how much you have had trouble falling asleep, staying asleep, or sleeping too much?	– All adolescents understood this item – *Trouble staying asleep means to wake up from sleep due to nightmares or bad dreams or due to thinking too much about their problems* – *People have difficulties to fall asleep or keep waking up from sleep (nindra bata bich bich ma byuijhirahane) when they have tension or when their body aches*
Poor appetite, weight loss, or overeating	In the past 2 weeks, how much have you felt like poor appetite, overeating than before or weight loss for no reason?	– Most adolescent understood this item – All adolescents suggested splitting this question into two to three questions—(a) poor appetite or overeating and (b) weight loss – Adolescents reported that people are intentionally trying to lose weight nowadays. As a result, they suggest changing the term “weight loss” to “getting thinner without any reason” (bina karan dublaudai jane or nachaahada nachaahadai dublaudai jane) *(bina karan dublaudai jane or nachaahada nachaahadai dublaudai jane.)* – *Loss of appetite means not wanting to eat from their heart-mind (man bata nai khana man nalagne) and people don’t want to eat when they are angry, stressed or depressed*
Feeling tired, or having little energy	In the past 2 weeks, how much have you felt like tired or like you are weak or have little energy?	– Most of the adolescent understood this item – *We feel tired from spending almost 12 h at school and the pressure of studying at both school and home. As a result, we feel weak if we don’t eat good, nutritious food*
Feeling bad about yourself/that you are a failure/letting people down	In the past 2 weeks, how often did you feel bad about yourself, or feel like you are a failure, or feel like you have let yourself or your family down? Your or your family’s respect or dignity has decreased because of you.	– Most of the adolescents reported that this question is too long, and it also consists of many ideas that created confusion – They suggested splitting it into multiple questions – *Sometimes I feel bad about myself, feels like a failure and has let my family down. I feel like this when I see my friends doing progress in their life while I am not, despite of my strong desire*
Trouble concentrating on things/school, work, reading or watching TV	In the past 2 weeks, how much have you had trouble in concentrating on things? (e.g. reading in school, doing homework)	– Most of the adolescents understood this item – *‘Watching TV’* was removed from the example because they said that ‘watching TV’ is the most enjoyable thing for adolescents. – They said that this item means *‘the eyes are there and the mind is in other places.* – *Sometime it is hard to concentrate due to tension, sadness; however, adolescents are able to keep their concentration on TV at any time though they have tension because watching TV is interesting thing to do for the adolescents*
Moving or speaking slowly/being fidgety or restless	In the past 2 weeks, how much did you feel like moving or speaking so slowly that other people could have noticed? *And* How much did you like being so restless that you were moving around a lot more than usual?	– Most of the adolescent did not understand the first part (i.e. walking or speaking slowly) but they understood the second part (being so restless) easily – They suggested splitting the item into two questions. – Adolescents understood that being restless is like ‘what to do, where to go, what to eat’ – *Sometime I also feel restless during examination time. People panic (aatinchan) and try to do things speedily while feeling restless*
Thoughts that you would be better off dead/hurting yourself	In the past 2 weeks, how often did you think or have idea like you would be better off dead or of physically hurting yourself in some way? (For example: cut own hand, jumping from tall place, bang own head on the wall, etc.)	– Some adolescent had difficulty in understanding this item – Adolescents said that thoughts of dying *(afu maru maru jasto soch bichar aaune)* means loss of self-esteem and not wanting to live in this world – Adolescents suggested to add ‘physically hurt’ *(sharirik rup ma hani wa chot puryaune)* to make the item clearer. – Adolescents think that people try to hurt themselves to show their grief to others

**Table 2:** GAD-7 original item, back-translation of Nepali items, and comments from adolescents

Original Items	Back translation of the final Nepali version	Comments
Feeling nervous, anxious or on edge	In the past 2 weeks, how much have you been feeling nervous or anxious?	– Most of the adolescents understood this item – Adolescents suggested to keep “nervous” as it is (i.e. in english language) because it is quite commonly used in Nepali – Both translators and adolescents suggested excluding “on edge” from Nepali translation because it is not common in Nepali – Adolescent reported that they feel nervous when they try to speak in front of many people or while attending interviews – *I feel anxious when my father and mother fight with each-other. I feel anxious that my neighbors may know it and it may also disturb them*
Not being able to stop or control worrying	In the past 2 weeks, how much have you felt like you are unable to control or manage your anxiety?	– Some adolescents had difficulty in understanding this item – Adolescent reported that it might be difficult for people with depression to control their worry – *I was not able to control my worry and anxiety when I had an accident*
Worrying too much about different things	In the past 2 weeks, how much have you been worried about so many things? (For example: worries about study, worries about exams, worries about family matters, etc.)	– Most of the adolescents interpreted this item differently in the beginning. They suggested including an example to clarify the meaning of “different things” – An example of “worries about study, worries about exams, worries about family matters, etc.,” has been added to clarify the term “different things” – Adolescents reported that this question means worry too much about something. *[Kunai kura ma atyadhik chinta garnu]*
Trouble relaxing	In past 2 weeks, how much have you been unable to sit relaxing because of fidgety in your mind?	– Some adolescents misunderstood this question in the beginning. They linked “trouble relaxing” with physical aspects – “Because of fidgety in your mind” has been added to clarify this question – *At the time of earthquake, we weren’t able to relax and stay calmly* – *If we need to do a lot of homework at once, we get tense and have difficult relaxing*
Being so restless that it is hard to sit still	In the past 2 weeks, how often have you been so restless that you were unable to sit or stay in one place?	– Most of the adolescents relate this item with previous item i.e. “trouble relaxing”. They thought that “trouble relaxing” and “being so restless that it is hard to sit still” are the same – Adolescents understood this as ‘very difficult from inside, not being able to sit in one place, walking here and there’ – *Restlessness occurs in your mind when tension is bothering you. It is a feeling of what to do now and where to go*
Becoming easily annoyed or irritable	In the past 2 weeks, how often do you feel upset or irritated by small things?	– Most of the adolescents could not understand this question in the beginning, but all understood it when it was simplified by replacing “easily” with “in small things” – Adolescents understood this item as ‘getting angry even in a small thing/issue’ – I feel so irritable when by father and mother call me while I am playing online games – It makes me irritable [*jhijo lagne*] when friends send messages while attending an online examination
Feeling afraid, as if something awful might happen	In the past 2 weeks, how much fear did you have in your mind that something bad would happen to you? (for example, fearing that you or your loved one or a family member will have an accident, get sick, or fail an exam)	– Almost all adolescents could not understand this question in the beginning but they understood when examples were added – Examples such as “fearing that you or your loved one or a family member may get an accident, or may get sick, or you may fail an exam etc.,” have been added to clarify the term ‘awful might happen’ – *When my father comes home drinking, there is a fear that something bad will happen at home*

The major adaptation made in both PHQ-A and GAD-7 included changing statements into questions, using a visual scale (glass scale) to maintain uniformity in responses and keeping a time frame (i.e. in the past two weeks) in the beginning of each item. We included a water glass response pictorial scale to maintain uniformity in assessing the severity of each symptom. This scale was developed through a transcultural translation process with children in Nepal (Kohrt et al. [18]). Adolescents in the adaptation phase found that using the water glass response scale helped them differentiate between different response options.

Other adaptations suggested by the adolescents included providing examples to clarify specific questions, dividing certain items into multiple questions, and ensuring that items are relevant to the local context by explaining the meaning of specific terminology or idioms. For example, adolescents mentioned that “watching TV” is one of their most enjoyable activities, so it was recommended to remove “watching TV” from PHQ-A item-7.

*Sometimes it is hard to concentrate due to tension or sadness. However, adolescents are able to maintain their concentration on TV at any time, even when they are feeling tense, because watching TV is an interesting activity for them.****Cognitive interview-8***.

Moreover, during the FGDs and CIs, most of the adolescents recommended splitting PHQ-A items 3, 5, 6, and 8 into multiple questions. They observed that these items encompassed multiple themes or ideas within a single question. For instance, during a cognitive interview, one adolescent mentioned experiencing sleep problems for several days. However, he pointed out that he couldn’t provide the same response to all three issues, such as having difficulty falling asleep, waking up during sleep, or feeling sleepier than before.

Similarly, during the FGDs and CIs, many adolescents suggested breaking down PHQ-A items 3, 5, 6, and 8 into multiple questions. They noticed that these items covered multiple themes or ideas within a single question. For example, during a cognitive interview, one adolescent mentioned having sleep problems for several days. However, he noted that he couldn’t give the same response to all three issues, such as difficulty falling asleep, waking up during sleep, or feeling sleepier than before.

*This question (PHQ-9 item3) contains three ideas/concepts, therefore, it makes hard to understand once at a time. Therefore, I suggest asking the first two ideas in a separate question, and last idea in another question–****Cognitive interview-9***.

Participants reported that there is no idiom or appropriate terminology for “feeling on edge” (GAD-7 item-1) in Nepali. Therefore, they suggested excluding this term from the Nepali translation. Additionally, an example of “writing, playing games, hanging out, using mobile” has been suggested to added clarifying the term “things” for GAD-7 item-3. Most participants associated “trouble relaxing” (GAD-7 item-4) with physical aspects, so this has been further clarified by adding “because of (feeling) fidgety in your mind”. Furthermore, the term “easily annoyed or irritable” in GAD-7 item-6 has been changed to “annoyed or irritable in small things”. An example has also been added in GAD-7 item-7 to clarify the term “awful” (Table 2).

### Phase-2 validation study results

Table 3 displays the socio-demographic characteristics of the participants in the validation study (Phase II). The majority of the participants were girls (55%). Their ages ranged from 12 to 19 years, with most falling between 15 and 19 years old (62%). The most common castes were Brahmin/Chhetri (53.1%) or Janajati (36.1%), and the majority practiced Hinduism (79.7%) and spoke Nepali at home (81.4%).

**Table 3:** Social demographic characteristics of the participants involved in the validation study

	Boys *N* = 186 %	Girls *N* = 227 %	Total *N* = 413 %
*Age*			
12–14	34.9	40.5	38.0
15–19	65.1	59.5	62.0
Mean (SD)	15.2 (2.1)	15.3 (2.1)	15.3 (2.1)
*Education*			
Up to grade 5	8.1	3.1	5.3
Grade 6 to 8	29.6	33.0	31.5
Grade 9 to 10	45.7	38.3	41.6
Grade 11 or more	16.7	25.6	21.6
*Caste*			
Brahmin/Chhetri	55.9	51.2	53.3
Janajati	33.4	38.3	36.1
Dalit	4.8	5.7	5.2
Others	5.9	4.8	5.4
*Marital status*			
Single	100	97.8	98.8
Married	0	2.2	1.2
*Religion*			
Hindu	77.4	81.5	79.7
Buddhist	15.1	16.3	15.7
Others	7.5	2.2	4.6
*Language spoken at home*			
Nepali	81.7	81.1	81.4
Newari	4.8	3.5	4.1
Tamang	8.6	11.5	10.2
Others	4.9	3.9	4.3
Total	45.0	55.0	100.0

#### Psychometric properties for PHQ-A

We presented the area under the curves (AUCs) for both PHQ-A and GAD-7 separately for girls, boys, younger, and older adolescents. The AUC of PHQ-A for boys was 0.81 (95% CI, 0.68–0.93), while it was 0.88 (95% CI, 0.82–0.93) for girls. Similarly, the AUC for younger adolescents was 0.92 (95% CI, 0.85–0.98), while it was 0.85 (95% CI, 0.79–0.92) for older adolescents.

Table 4 presents the psychometric properties of the adapted PHQ-A compared with the K-SADS depression module. A cut-off score for the overall sample of > = 11, yields sensitivity of 0.90 and specificity of 0.71. The positive predictive value for the cut-off score of > = 11 was 0.31 and 0.98. The results suggested different cut-off scores for younger and older adolescents. A cutoff score for younger adolescents (12 to 14 years) is > = 13, with a sensitivity of 0.93 and specificity of 0.80. The PPV and NPV for > = 13 for younger adolescents were 0.33 and 0.99. On the other hand, a cut-off score for older adolescents was > = 11, with a sensitivity of 0.89 and specificity of 0.70, and PPV of 0.32 and NPV of 0.97 (Table 2). Similarly, the results also suggested different cut-off scores for boys and girls. A cut-off score of > = 11 with a sensitivity of 0.73 and specificity of 0.78 has been suggested for boys. The PPV and NPV for the > = 11 cut-off for boys were 0.17 and 0.97. Similarly, a cut-off score for girls of > = 13 has a sensitivity of 0.85 and specificity of 0.73. The cut-off score of > = 13 has a PPV of 0.40 and NPV of 0.96 (supplementary table-1).

**Table 4:** Validation psychometrics of the PHQ-A comparison with the K-SADS

	Cut-off score	Sensitivity	Specificity	PPV	NPV	PLR	NLR	Diagnostic OR	Youden’s Index (J)	TP (%)	TN (%)	FP (%)	FN (%)	Accurately classified (%)
Total sample	≥ 8	0.90	0.57	0.23	0.98	2.12	0.17	12.43	0.48	0.11	0.50	0.37	0.01	0.62
	≥ 9	0.90	0.63	0.25	0.98	2.42	0.16	15.47	0.53	0.11	0.55	0.33	0.01	0.66
	≥ 10	0.90	0.69	0.29	0.98	2.86	0.14	20.01	0.59	0.11	0.60	0.28	0.01	0.71
	≥ 11	0.90	0.71	0.31	0.98	3.14	0.14	22.82	0.61	0.11	0.62	0.25	0.01	0.74
	≥ 12	0.80	0.75	0.31	0.96	3.23	0.26	12.39	0.56	0.10	0.66	0.22	0.02	0.76
	≥ 13	0.78	0.79	0.34	0.96	3.69	0.27	13.46	0.57	0.10	0.69	0.19	0.03	0.79
	≥ 14	0.75	0.83	0.38	0.96	4.35	0.31	14.14	0.57	0.09	0.73	0.15	0.03	0.82
12–14 years old	≥ 10	0.93	0.73	0.27	0.99	3.49	0.09	38.32	0.67	0.09	0.66	0.24	0.01	0.75
	≥ 11	0.93	0.74	0.27	0.99	3.58	0.09	39.73	0.67	0.09	0.67	0.24	0.01	0.76
	≥ 12	0.93	0.77	0.30	0.99	4.14	0.09	48.13	0.71	0.09	0.70	0.20	0.01	0.79
	≥ 13	0.93	0.80	0.33	0.99	4.73	0.08	57.00	0.74	0.09	0.73	0.18	0.01	0.82
	≥ 14	0.87	0.82	0.33	0.98	4.73	0.16	29.00	0.68	0.08	0.74	0.17	0.01	0.82
	≥ 15	0.87	0.84	0.36	0.98	5.35	0.16	33.63	0.70	0.08	0.76	0.15	0.01	0.84
	≥ 16	0.80	0.86	0.38	0.98	5.68	0.23	24.40	0.66	0.08	0.78	0.13	0.02	0.85
15–19 years old	≥ 8	0.89	0.55	0.25	0.97	2.00	0.20	9.96	0.44	0.13	0.48	0.38	0.02	0.60
	≥ 9	0.89	0.60	0.27	0.97	2.25	0.18	12.23	0.49	0.13	0.52	0.34	0.02	0.64
	≥ 10	0.89	0.65	0.30	0.97	2.57	0.17	15.16	0.54	0.13	0.56	0.3	0.02	0.69
	≥ 11	0.89	0.70	0.32	0.97	2.92	0.16	18.27	0.58	0.13	0.6	0.26	0.02	0.72
	≥ 12	0.75	0.74	0.32	0.95	2.84	0.34	8.38	0.49	0.11	0.63	0.23	0.04	0.74
	≥ 13	0.72	0.78	0.35	0.94	3.24	0.36	9.07	0.50	0.10	0.67	0.19	0.04	0.77
	≥ 14	0.69	0.84	0.41	0.94	4.24	0.37	11.62	0.53	0.10	0.72	0.14	0.04	0.82

PHQ-A  Patients Health Questionnaire for Adolescents; GAD-7   Generalized Anxiety Disorder; K-SADS  Kiddie Schedule for Affective Disorders and Schizophrenia; PPV  Positive Predictive Value; NPV Negative Predictive Value; PLR   Positive Likelihood Ratio; NLR  Negative Likelihood Ratio

TP = True Positive; TN = True Negative; FP = False Positive; FN = False Negative

### Psychometric properties for GAD-7

The AUC of GAD-7 was 0.80 (95% CI, 0.73–0.87) for younger adolescents and 0.76 (95% CI, 0.70–0.82) for older adolescents. For boys, the AUC was 0.77 (95% CI, 0.69–0.85) and slightly lower for girls at 0.74 (95% CI, 0.68–0.81).

Table 5 presents the psychometric properties of GAD-7 for the overall sample, boys, girls, and younger and older adolescents separately. A cut-off score for the overall sample of > = 7 yields a sensitivity of 0.83 and specificity of 0.61. The PPV and NPV for the cut-off score of > = 7 were 0.41 and 0.92. Acut-off score for both younger and older adolescents was 8, with the same sensitivity (0.70 for both groups) but slightly different specificities (0.67 for younger and 0.71 for older adolescents), PPVs (0.39 for younger and 0.50 for older adolescents), and NPVs (0.89 for younger and 0.85 for older adolescents). The results suggested different cut-off scores for boys and girls. A cut-off score of > = 7 with a sensitivity of 0.75 and specificity of 0.71 has been suggested for boys. The PPV and NPV for the > = 7 cut-off for boys were 0.31 and 0.94. Similarly, a cut-off score for girls of > = 8 yields a sensitivity of 0.76 and specificity of 0.61. The cut-off score of > = 8 has a PPV of 0.51 and NPV of 0.83 (supplementary table-1).

**Table 5:** Validation psychometrics of the GAD-7 from comparison with the K-SADS

	Cut-off score	Sensitivity	Specificity	PPV	NPV	PLR	NLR	Diagnostic OR	Youden’s Index (J)	TP (%)	TN (%)	FP (%)	FN (%)	Accurately classified (%)
Total Sample	≥ 4	0.95	0.42	0.35	0.96	1.65	0.12	14.31	0.38	0.23	0.32	0.43	0.01	0.55
	≥ 5	0.93	0.50	0.38	0.96	1.86	0.14	13.48	0.43	0.23	0.38	0.38	0.02	0.61
	≥ 6	0.86	0.55	0.38	0.92	1.90	0.25	7.58	0.41	0.21	0.41	0.34	0.03	0.62
	≥ 7	0.83	0.61	0.41	0.92	2.14	0.27	7.85	0.44	0.21	0.46	0.29	0.04	0.67
	≥ 8	0.75	0.69	0.45	0.90	2.47	0.35	7.00	0.45	0.19	0.52	0.23	0.06	0.71
	≥ 9	0.71	0.72	0.45	0.88	2.52	0.41	6.18	0.43	0.17	0.54	0.21	0.07	0.72
	≥ 10	0.66	0.75	0.46	0.87	2.59	0.46	5.62	0.40	0.16	0.56	0.19	0.08	0.72
12–14 years old	≥ 5	0.78	0.46	0.30	0.88	1.45	0.47	3.09	0.24	0.18	0.37	0.43	0.01	0.55
	≥ 6	0.73	0.53	0.31	0.87	1.56	0.51	3.07	0.26	0.17	0.43	0.38	0.03	0.60
	≥ 7	0.73	0.57	0.33	0.88	1.70	0.47	3.60	0.30	0.17	0.46	0.34	0.03	0.63
	≥ 8	0.70	0.67	0.39	0.89	2.16	0.44	4.90	0.38	0.17	0.54	0.26	0.03	0.71
	≥ 9	0.68	0.71	0.41	0.88	2.36	0.45	5.21	0.39	0.16	0.57	0.23	0.04	0.73
	≥ 10	0.62	0.74	0.41	0.87	2.37	0.51	4.63	0.36	0.15	0.59	0.21	0.05	0.74
	≥ 11	0.57	0.79	0.44	0.86	2.65	0.55	4.81	0.35	0.13	0.63	0.17	0.06	0.76
15–19 years old	≥ 5	0.90	0.52	0.44	0.92	1.88	0.20	9.51	0.42	0.26	0.38	0.34	0.02	0.64
	≥ 6	0.83	0.56	0.44	0.89	1.88	0.30	6.18	0.39	0.24	0.40	0.32	0.04	0.64
	≥ 7	0.79	0.64	0.48	0.88	2.19	0.33	6.71	0.43	0.23	0.46	0.26	0.05	0.69
	≥ 8	0.70	0.71	0.50	0.85	2.40	0.42	5.70	0.41	0.20	0.51	0.21	0.08	0.71
	≥ 9	0.65	0.72	0.50	0.83	2.36	0.48	4.87	0.37	0.18	0.52	0.20	0.09	0.71
	≥ 10	0.61	0.75	0.51	0.82	2.45	0.52	4.73	0.36	0.17	0.54	0.18	0.11	0.71
	≥ 11	0.56	0.78	0.52	0.81	2.58	0.56	4.58	0.34	016	0.57	0.16	0.12	0.72

PHQ-A Patients Health Questionnaire for Adolescents; GAD-7 Generalized Anxiety Disorder; K-SADS Kiddie Schedule for Affective Disorders and Schizophrenia; PPV Positive Predictive Value; NPV Negative Predictive Value; PLR Positive Likelihood Ratio; NLR Negative Likelihood Ratio

TP = True Positive; TN = True Negative; FP = False Positive; FN = False Negative

### Adjustments for population prevalence

**Fig. 2:**
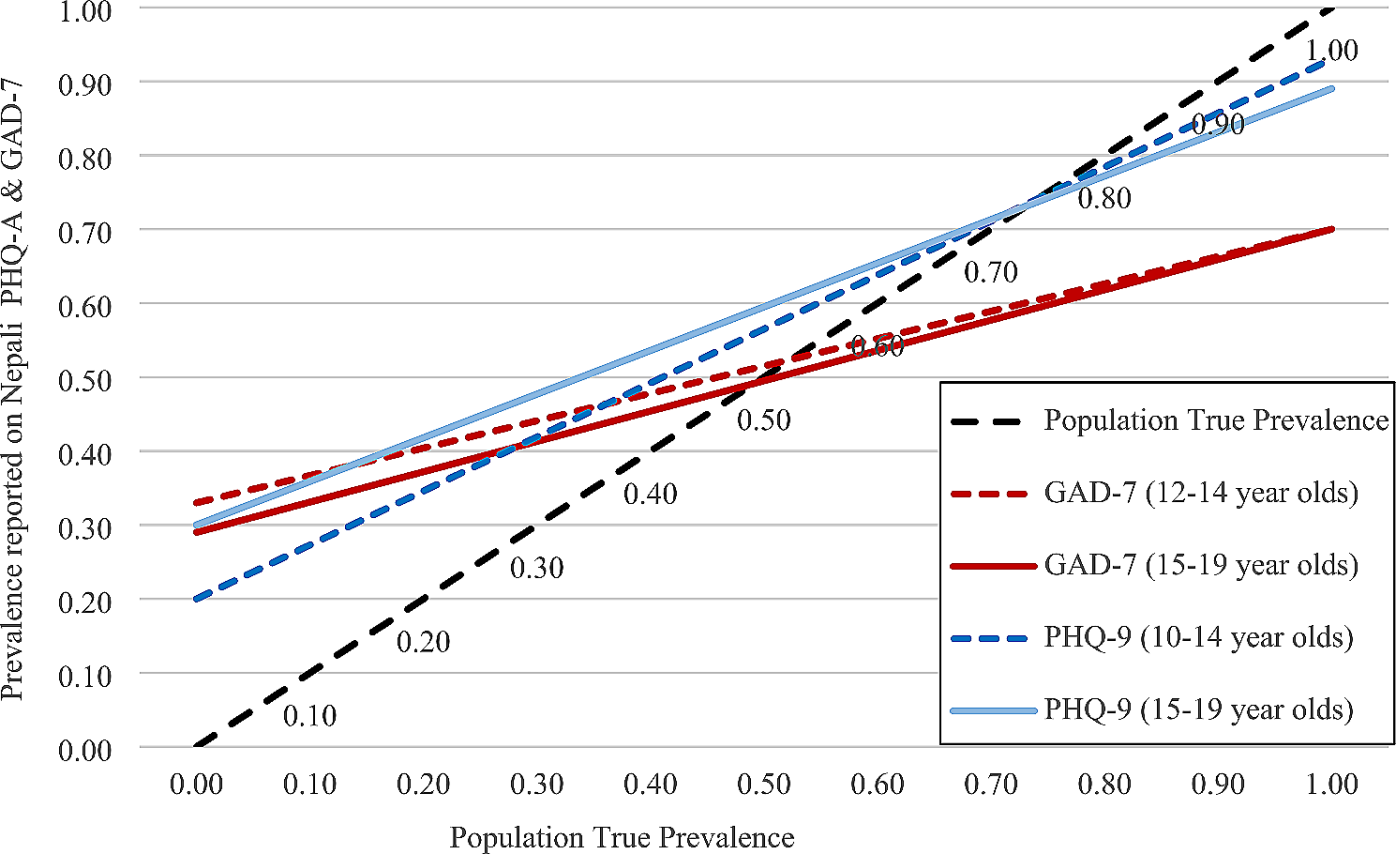
Application of the validation results: adjustments for population level prevalence

Figure 2 shows the adjusted prevalence rates for adolescent depression and anxiety at the population level. These rates were calculated using the psychometric properties obtained in this study. We adjusted the false positive (FP) and false negative (FN) rates in the current sample to calculate the true prevalence in the population. For instance, if a prevalence rate of 40% is found for depression on the PHQ-A, the estimated true prevalence rate in younger populations is closer to 25% and for older adolescents it would be below 20%.

### Item level analysis

The internal consistency reliability of the PHQ-A (Cronbach’s alpha) was 0.914. Table 6 shows the mean scores of each PHQ-A item for adolescents diagnosed with depression and those without a diagnosis. The results indicate that the PHQ-A items have excellent discriminant ability between depressed and non-depressed adolescents. The mean scores of all PHQ-A items were significantly higher (*P* < .001) among adolescents with depression compared to those without a diagnosis. Similarly, the mean scores of all PHQ-A items were significantly higher among younger and older adolescents with depression compared to their counterparts without a diagnosis.

**Table 6:** Discriminant ability of PHQ-A items for adolescents with and without diagnosis on K-SADS

Item# Description	No diagnosis *N* = 241 Mean (SD)	Any depression diagnosis *N* = 51 Mean (SD)	t-test	p-value
PHQ-A1 Feeling down, depressed, irritable, or hopeless	0.80 (0.83)	2.06 (0.79)	− 9.95	< 0.001
PHQ-A2 Little interest or pleasure in doing things	0.68 (0.89)	1.78 (0.81)	− 8.18	< 0.001
PHQ-A3 Trouble falling asleep, or staying asleep or sleeping too much	0.58 (0.82)	1.67 (0.93)	− 8.39	< 0.001
PHQ-A4 Poor appetite, weight loss, or overeating	0.52 (0.80)	1.90 (0.86)	− 11.13	< 0.001
PHQ-A5 Feeling tired, or having little energy	0.71 (0.91)	2.14 (0.83)	− 10.32	< 0.001
PHQ-A6 Feeling bad about yourself /that you are a failure/letting people down	0.68 (0.95)	2.14 (0.85)	− 10.13	< 0.001
PHQ-A7 Trouble concentrating on things/school, work, reading	0.84 (0.96)	2.10 (0.83)	− 9.57	< 0.001
PHQ-A8 Moving or speaking slowly/being fidgety or restless	0.41 (0.79)	1.49 (1.01)	− 7.23	< 0.001
PHQ-A9 Thoughts that you would be better off dead/hurting yourself	0.33 (0.68)	1.72 (1.16)	− 8.14	< 0.001
***Age 12 to 14***	***N*** ** = 97**	***N*** ** = 15**		
PHQ-A1 Feeling down, depressed, irritable, or hopeless	0.86 (0.78)	2.20 (0.78)	− 6.24	< 0.001
PHQ-A2 Little interest or pleasure in doing things	0.76 (0.90)	1.73 (0.88)	− 3.90	< 0.001
PHQ-A3 Trouble falling asleep, or staying asleep or sleeping too much	0.54 (0.77)	2.00 (0.93)	− 6.71	< 0.001
PHQ-A4 Poor appetite, weight loss, or overeating	0.61 (0.82)	2.27 (0.80)	− 7.29	< 0.001
PHQ-A5 Feeling tired, or having little energy	0.72 (0.85)	2.47 (0.83)	− 7.41	< 0.001
PHQ-A6 Feeling bad about yourself/that you are a failure/letting people down	0.61 (0.82)	2.60 (0.63)	− 8.95	< 0.001
PHQ-A7 Trouble concentrating on things/school, work, reading	0.89 (0.97)	2.33 (0.90)	− 5.44	< 0.001
PHQ-A8 Moving or speaking slowly/being fidgety or restless	0.49 (0.84)	1.87 (0.99)	− 5.73	< 0.001
PHQ-A9 Thoughts that you would be better off dead/hurting yourself	0.34 (0.71)	2.43 (0.94)	− 9.85	< 0.001
***Age group 15–19 years***	***N *** ** = 144**	***N *** **= 36**		
PHQ-A1 Feeling down, depressed, irritable, or hopeless	0.76 (0.86)	2.00 (0.79)	− 8.22	< 0.001
PHQ-A2 Little interest or pleasure in doing things	0.63 (0.88)	1.81 (0.79)	− 7.34	< 0.001
PHQ-A3 Trouble falling asleep, or staying asleep or sleeping too much	0.60 (0.86)	1.53 (0.91)	− 5.68	< 0.001
PHQ-A4 Poor appetite, weight loss, or overeating	0.46 (0.77)	1.75 (0.84)	− 8.80	< 0.001
PHQ-A5 Feeling tired, or having little energy	0.70 (0.95)	2.00 (0.79)	− 8.42	< 0.001
PHQ-A6 Feeling bad about yourself/that you are a failure / letting people down	0.73 (1.03)	1.94 (0.86)	− 7.28	< 0.001
PHQ-A7 Trouble concentrating on things/school, work, reading	0.81 (0.96)	2.00 (0.79)	− 7.74	< 0.001
PHQ-A8 Moving or speaking slowly/being fidgety or restless	0.35 (0.74)	1.33 (0.99)	− 5.62	< 0.001
PHQ-A9 Thoughts that you would be better off dead/hurting yourself	0.33 (0.65)	1.44 (1.13)	− 5.67	< 0.001

Supplemental Table 4 showed that there was no significant difference in the mean score of PHQ-9 item-9 (thoughts of being better off dead/hurting yourself) between boys with and without depression. However, the mean scores of all PHQ-9 items were significantly higher among girls with anxiety (supplemental Table 4).

The internal consistency reliability for GAD-7 (Cronbach’s alpha) was 0.907. Table 7 shows that the mean scores of all GAD-7 items were significantly higher among adolescents diagnosed with generalized anxiety disorder than those without any diagnosis. Similarly, the mean scores of all GAD-7 items were also significantly higher among younger and older adolescents with GAD compared to their counterparts without any diagnosis (Table 7).

**Table 7:** Discriminant ability of GAD-7 items for adolescents with and without diagnosis on the K-SADS

Item# Description	No diagnosis *N* = 241 Mean (SD)	Any GAD diagnosis *N* = 102 Mean (SD)	t-test	p-value
GAD-1 Feeling nervous. anxious or on edge	0.73 (0.85)	1.77 (0.78)	− 10.61	< 0.001
GAD-2 Not being able to stop or control worrying	0.53 (0.87)	1.54 (0.97)	− 9.05	< 0.001
GAD-3 Worrying too much about different things	0.68 (0.96)	1.74 (0.92)	− 9.45	< 0.001
GAD-4 Trouble relaxing	0.48 (0.78)	1.57 (0.97)	− 10.03	< 0.001
GAD-5 So restless that it is hard to sit still	0.47 (0.85)	1.39 (1.01)	− 8.09	< 0.001
GAD-6 Easily annoyed / irritable	1.03 (1.05)	1.90 (0.98)	− 7.19	< 0.001
GAD-7 Feeling afraid as if something awful might happen	0.67 (0.95)	1.38 (0.96)	− 6.25	< 0.001
***Age 12 to 14 years***	***N*** **= 97**	***N*** ** = 31**		
GAD-1 Feeling nervous. anxious or on edge	0.77 (0.81)	1.90 (0.70)	− 6.97	< 0.001
GAD-2 Not being able to stop or control worrying	0.69 (1.00)	1.71 (0.90)	− 5.04	< 0.001
GAD-3 Worrying too much about different things	0.68 (0.87)	2.10 (0.87)	− 7.87	< 0.001
GAD-4 Trouble relaxing	0.47 (0.75)	1.68 (0.95)	− 6.47	< 0.001
GAD-5 So restless that it is hard to sit still	0.56 (0.89)	1.48 (1.06)	− 4.82	< 0.001
GAD-6 Easily annoyed / irritable	1.10 (1.05)	2.10 (1.08)	− 4.57	< 0.001
GAD-7 Feeling afraid as if something awful might happen	0.74 (0.93)	1.65 (1.05)	− 4.57	< 0.001
***Age 15–19 years***	***N*** ** = 144**	***N*** ** = 71**		
GAD-1 Feeling nervous. anxious or on edge	0.71 (0.88)	1.72 (0.81)	− 8.13	< 0.001
GAD-2 Not being able to stop or control worrying	0.42 (0.75)	1.46 (1.00)	− 7.77	< 0.001
GAD-3 Worrying too much about different things	0.67 (1.02)	1.58 (0.91)	− 6.35	< 0.001
GAD-4 Trouble relaxing	0.49 (0.80)	1.52 (0.98)	− 7.70	< 0.001
GAD-5 So restless that it is hard to sit still	0.42 (0.82)	1.35 (0.99)	− 6.91	< 0.001
GAD-6 Easily annoyed / irritable	0.98 (1.05)	1.82 (0.93)	− 5.72	< 0.001
GAD-7 Feeling afraid as if something awful might happen	0.63 (0.96)	1.27 (0.96)	− 4.62	< 0.001

Supplemental Table 5 shows that there was no significant difference in the mean score of GAD-7 item 7 (feeling afraid as if something awful might happen) between boys with and without depression. However, the mean scores of all GAD-7 items were significantly higher among girls with anxiety (Supplemental Table 5).

## Discussion

This study translated, culturally adapted, and validated the PHQ-A and GAD-7 for Nepali-speaking adolescents aged 12 to 19 years, following the methods outlined in UNICEF’s MMAPP protocol (Carvajal-Velez et al. [4]). These methods are based on the systematic process recommended for cross-cultural research (Kohrt et al. [18]; van Ommeren et al. [38]). Key adaptations to both the PHQ-A and GAD-7 included changing statements to question form, using a visual scale (e.g., a glass with water) to standardize responses, adding a timeframe to each item, and simplifying the language with local idioms of distress to fit the local context. Examples were also added to clarify the meaning of multiple items in the PHQ-A and GAD-7. Additionally, some words and terminologies were changed to make them more relevant to the local context. For example, “weight loss” was replaced by “getting thinner without intention” or “losing weight for no reason.” Similarly, the term “trouble relaxing” was further clarified by adding “because of fidgety in your mind,” as adolescents understood “trouble relaxing” as a physical problem.

The adolescents who participated in the adaptation process made a key comment about the need to split an item with multiple ideas into different questions. They noted that participants tend to remember the last part of the question and respond accordingly. For example, they suggested splitting PHQ-A item-4 into three separate questions: poor appetite, overeating, and weight loss for no reason, as they believed these are distinct ideas that should not be combined into one question. Most of the adolescents also commented that each item should only include one idea, while commonly used mental health tools often include multiple ideas in one item (known as double-barrel questions). Another interesting finding is that the examples provided by participants in this study differed from those in previous studies. For instance, in the past, visiting a maternal uncle’s house was reported as the most enjoyable activity among adolescents (Kohrt et al. [18]), but none of the adolescents in this study mentioned it. Instead, they reported enjoying activities such as watching TV or playing games on mobile phones. This suggests that examples of enjoyable activities change over time, and it is important for tools to be adaptable to reflect adolescents’ current experiences, which may differ from those of previous generations.

Adolescents linked their mental health problems with tension/stress (*tanab*) and bodily complaints. They explained how tension affects their bodies and daily lives. Many existing mental health assessment tools do not include items related to bodily complaints, which are important indicators for accurately detecting mental health problems. In Nepal, the understanding and expression of mental health and psychosocial problems is complex among the various ethnic and cultural groups. Generally, people express their mental health problems through their bodies, and the heart-mind (*man*) is believed to be an organ of emotion and feeling, while the brain-mind (*dimaag*) is considered an organ of cognition (Kohrt and Harper [17]). Therefore, it is important to include physical or bodily complaints in mental health assessment tools to capture the specific idioms of distress in different contexts.

The results showed that the culturally adapted Nepali version of the PHQ-A had fair sensitivity, specificity, diagnostic agreement, and overall accuracy for adolescents in Nepal. A cut-off score of 11 could be for general use, but different cut-off scores should be used based on intended use of the tool (e.g., is higher sensitivity, specificity, PPV, or NPV desired), then the cut-off should be adjusted accordingly (Kohrt and Kaiser [19]; Conflict and health). Different cut-offs can be used for specific groups such as younger or older adolescents, boys or girls, and clinical or community-based samples. Adolescents with depression had significantly higher mean scores in all individual symptom PHQ-A items, demonstrating discriminant validity of individual depression symptoms. The sensitivity and specificity for the cut-off score of 11 were 0.90 and 0.71, respectively. Our sensitivity results are consistent with or better than those summarized in a recent systematic review of 29 validation studies of the PHQ-9 (Carroll et al. [3]), which reported a combined sensitivity and specificity at a cut-off score of 10 or above as 0.88 and 0.85, respectively (Levis et al. [21]). However, the specificity was considerably lower for our study compared the systematic review findings, which illustrates high rates of false positives when using the tool with a similar cut-off in Nepal. The cut-off score recommended in our study (11 or more) is consistent with a study conducted in Chile. As with the systematic review findings, the specificity reported in our study (0.71) was much lower than that of the Chilean study (0.83) (Borghero et al. [2]). Similarly, a cut-off score of 11 with a sensitivity of 89.5 and specificity of 77.5 has been recommended for adolescents in the USA (Richardson et al. [30]), while a cut-off score of 9 with a sensitivity of 89.0% and specificity of 70.0% has been recommended for Swahili-speaking adolescents in Kenya, with the caveat that those authors also called for caution because of the high false positive rates (Tele et al. [37]). In Mozambique, a cut-off score of 8 with a sensitivity of 0.78 and specificity of 0.80 has been used for adolescents (Lovero et al. [22]).

The GAD-7 showed strong internal consistency with a Cronbach’s alpha of 0.907. It has acceptable sensitivity (0.83) but only poor specificity (0.61) for a cut-off score of 7. Despite performing less optimally compared to the PHQ-A, the item analysis of all GAD-7 items demonstrated individual item discriminant ability to differentiate adolescents with generalized anxiety disorder from those without any diagnosis. However, due to limited data on the validation of the GAD-7 among adolescents, comparisons are restricted. Our results are similar to a validation study conducted in South Africa, where a cut-off score of 6 or more was recommended with a sensitivity of 0.75 and specificity of 0.67, also noting high false positive rates in that study (Marlow et al. [23]).

The results of this study could have several implications for accurately collecting data on the prevalence of depression and anxiety among adolescents in Nepal. First, the PHQ-A and GAD-7 were translated and adapted using a systematic process involving mental health experts and adolescents themselves. These tools use context-specific and non-stigmatized language, which may help participants share their problems and experiences more openly. Second, the study also recommended different cut-off scores for older/younger adolescents and boys/girls, which could improve the accuracy and reliability of the data for different sub-groups. Additionally, the results indicated that both PHQ-A and GAD-7 have high false positive rates and low PPV, so caution should be used when interpreting the results for reporting prevalence rates or for identifying adolescents to refer for interventions. To address this for future studies, we recommend adjusting identified rates by using the psychometric properties from this validation (as shown in Fig. 1 above). For clinical purposes and intervention studies, we strongly recommend conducting a clinical interview to confirm depression or anxiety before proceeding with an intervention given the low PPV.

The study has some limitations. The data collection for the validation study occurred from September 1, 2021, to December 21, 2021, during a period when some COVID-19 restrictions were still in effect. The results of the study may have been influenced by the ongoing pandemic and its stressors. Similarly, the participants in this study were recruited from schools and the outpatient department of the Kanti Children’s Hospital, so the study is lacking a community sample. Finally, as adolescents from certain language-speaking communities, such as Maithili, Bhojpuri, and Tharu, were underrepresented in the study, the findings may not accurately represent these caste/ethnic groups.

## Conclusion

The study addressed the current lack of systematically translated, adapted, and validated tools for measuring adolescent depression and anxiety in Nepal. The results showed that both the PHQ-A and GAD-7 are reliable tools for detecting and screening depression and anxiety among adolescents. Moreover, the approach to estimate true prevalence rate in the population can help policymakers and planners allocate resources to address mental health issues in this population.

### Electronic supplementary material

Below is the link to the electronic supplementary material.

Supplementary Material 1

## Data Availability

Interested parties may notify the investigators of their interest in collaboration, including access to the data-set analyzed here, through the following email: luitelnp@gmail.com.

## Abbreviations

AUC: Area under the curve
CI: Cognitive interviews
CIDT: Community informant detection tool
DSM-5: Diagnostic and Statistical Manual of Mental Disorders
FGD: Focus group discussion
GAD-7: Generalized anxiety disorder
K-SADS: Kiddie Schedule for Affective Disorders and Schizophrenia
LMIC: Low and Middle Income Countries
MMAPP: Measuring mental health among adolescents and young people at the population level
NPV: Negative predictive value
PHQ-A: Patient Health Questionnaire adapted for adolescents
PPV: Positive predictive value
UNICEF: United Nations Children’s Fund
