# Supplementary material for: Translation, cultural adaptation and validation of Patient Health Questionnaire and generalized anxiety disorder among adolescents in Nepal
Source: Child Adolesc Psychiatry Ment Health. 2024 Jun 19;18:74. doi: 10.1186/s13034-024-00763-7 (PMC11188246; doi:10.1186/s13034-024-00763-7)
Supplement: Supplementary file 1 — Supplementary Material 1 [file 13034_2024_763_MOESM1_ESM.docx]

Table 1 validation psychometrics of the PHQ-A comparison with the K-SADS

|  | Cut-off score | Sensitivity | Specificity | PPV | NPV | PLR | NLR | Diagnostic OR | Youden’s Index (*J*) | TP  (%) | TN  (%) | FP  (%) | FN  (%) | Accurately classified % (%) |
| --- | --- | --- | --- | --- | --- | --- | --- | --- | --- | --- | --- | --- | --- | --- |
| Boys | >=8 | 0.73 | 0.66 | 0.12 | 0.97 | 2.16 | 0.41 | 5.24 | 0.39 | 0.04 | 0.62 | 0.32 | 0.02 | 0.67 |
|  | >=9 | 0.73 | 0.71 | 0.14 | 0.98 | 2.55 | 0.38 | 6.67 | 0.44 | 0.04 | 0.67 | 0.27 | 0.02 | 0.72 |
|  | >=10 | 0.73 | 0.75 | 0.15 | 0.98 | 2.89 | 0.36 | 7.94 | 0.48 | 0.04 | 0.70 | 0.24 | 0.02 | 0.75 |
|  | >=11 | 0.73 | 0.78 | 0.17 | 0.98 | 3.35 | 0.35 | 9.61 | 0.51 | 0.04 | 0.74 | 0.20 | 0.02 | 0.78 |
|  | >=12 | 0.64 | 0.81 | 0.17 | 0.97 | 3.28 | 0.45 | 7.26 | 0.44 | 0.04 | 0.76 | 0.18 | 0.02 | 0.80 |
|  | >=13 | 0.55 | 0.85 | 0.19 | 0.97 | 3.67 | 0.53 | 6.88 | 0.40 | 0.03 | 0.80 | 0.14 | 0.03 | 0.83 |
|  | >=14 | 0.55 | 0.89 | 0.23 | 0.97 | 4.77 | 0.51 | 9.30 | 0.43 | 0.03 | 0.83 | 0.11 | 0.03 | 0.87 |
|  |  |  |  |  |  |  |  |  |  |  |  |  |  |  |
| Girls | >=10 | 0.95 | 0.63 | 0.35 | 0.98 | 2.54 | 0.08 | 31.76 | 0.58 | 0.17 | 0.52 | 0.31 | 0.01 | 0.68 |
|  | >=11 | 0.95 | 0.65 | 0.37 | 0.98 | 2.69 | 0.08 | 34.83 | 0.60 | 0.17 | 0.53 | 0.29 | 0.01 | 0.70 |
|  | >=12 | 0.85 | 0.70 | 0.38 | 0.96 | 2.84 | 0.21 | 13.26 | 0.55 | 0.15 | 0.58 | 0.25 | 0.03 | 0.73 |
|  | >=13 | 0.85 | 0.73 | 0.40 | 0.96 | 3.12 | 0.21 | 15.11 | 0.58 | 0.15 | 0.60 | 0.22 | 0.03 | 0.75 |
|  | >=14 | 0.80 | 0.78 | 0.43 | 0.95 | 3.56 | 0.26 | 13.81 | 0.58 | 0.14 | 0.64 | 0.19 | 0.04 | 0.78 |
|  | >=15 | 0.75 | 0.81 | 0.46 | 0.94 | 4.01 | 0.31 | 13.03 | 0.56 | 0.13 | 0.67 | 0.15 | 0.04 | 0.80 |
|  | >=16 | 0.70 | 0.87 | 0.54 | 0.93 | 5.45 | 0.34 | 15.85 | 0.57 | 0.12 | 0.72 | 0.11 | 0.05 | 0.84 |

Table 2 validation psychometrics of the GAD-7 from comparison with the K-SADS

|  | Cut-off score | Sensitivity | Specificity | PPV | NPV | PLR | NLR | Diagnostic OR | Youden’s Index (*J*) | TP  (%) | TN  (%) | FP  (%) | FN  (%) | Accurately classified (%) |
| --- | --- | --- | --- | --- | --- | --- | --- | --- | --- | --- | --- | --- | --- | --- |
| Boys | >=4 | 0.89 | 0.53 | 0.25 | 0.97 | 1.88 | 0.20 | 9.22 | 0.42 | 0.13 | 0.45 | 0.40 | 0.02 | 0.35 |
|  | >=5 | 0.89 | 0.59 | 0.28 | 0.97 | 2.20 | 0.18 | 12.24 | 0.49 | 0.13 | 0.51 | 0.34 | 0.02 | 0.46 |
|  | >=6 | 0.79 | 0.66 | 0.39 | 0.95 | 2.30 | 0.33 | 7.06 | 0.44 | 0.12 | 0.56 | 0.29 | 0.03 | 0.51 |
|  | >=7 | 0.75 | 0.71 | 0.31 | 0.94 | 2.58 | 0.35 | 7.30 | 0.46 | 0.11 | 0.60 | 0.25 | 0.04 | 0.58 |
|  | >=8 | 0.64 | 0.77 | 0.33 | 0.92 | 2.82 | 0.46 | 6.10 | 0.42 | 0.10 | 0.66 | 0.19 | 0.05 | 0.64 |
|  | >=9 | 0.61 | 0.79 | 0.34 | 0.92 | 2.91 | 0.50 | 5.85 | 0.40 | 0.09 | 0.67 | 0.18 | 0.06 | 0.68 |
|  | >=10 | 0.57 | 0.81 | 0.35 | 0.91 | 3.03 | 0.53 | 5.69 | 0.38 | 0.09 | 0.69 | 0.16 | 0.06 | 0.72 |
|  |  |  |  |  |  |  |  |  |  |  |  |  |  |  |
| Girls | >=5 | 0.90 | 0.40 | 0.44 | 0.88 | 1.50 | 0.25 | 5.97 | 0.30 | 0.31 | 0.27 | 0.41 | 0.02 | 0.58 |
|  | >=6 | 0.85 | 0.43 | 0.44 | 0.85 | 1.49 | 0.35 | 4.30 | 0.28 | 0.29 | 0.29 | 0.38 | 0.04 | 0.58 |
|  | >=7 | 0.83 | 0.51 | 0.47 | 0.85 | 1.68 | 0.34 | 4.90 | 0.33 | 0.28 | 0.34 | 0.33 | 0.04 | 0.63 |
|  | >=8 | 0.76 | 0.61 | 0.51 | 0.83 | 1.98 | 0.39 | 5.12 | 0.38 | 0.26 | 0.41 | 0.26 | 0.07 | 0.67 |
|  | >=9 | 0.71 | 0.65 | 0.51 | 0.81 | 2.02 | 0.44 | 4.54 | 0.36 | 0.24 | 0.44 | 0.24 | 0.08 | 0.68 |
|  | >=10 | 0.66 | 0.68 | 0.52 | 0.79 | 2.07 | 0.50 | 4.17 | 0.34 | 0.22 | 0.46 | 0.22 | 0.10 | 0.68 |
|  | >=11 | 0.60 | 0.73 | 0.53 | 0.78 | 2.19 | 0.55 | 3.96 | 0.33 | 0.20 | 0.49 | 0.19 | 0.12 | 0.69 |

Table 3

|  |  |  |  |  |  |  |  |  |  |  |  |  |
| --- | --- | --- | --- | --- | --- | --- | --- | --- | --- | --- | --- | --- |
| GAD-7 (12-14 year olds),  Cut-off ≥8, Sensitivity=0.70, Specificity=0.67 | Screening Tool Detected Prevalence | 0.33 | 0.37 | 0.40 | 0.44 | 0.48 | 0.52 | 0.55 | 0.59 | 0.63 | 0.66 | 0.70 |
|  | Difference between true prevalence and detected prevalence | 0.33 | 0.27 | 0.20 | 0.14 | 0.08 | 0.01 | -0.05 | -0.11 | -0.17 | -0.24 | -0.30 |
|  | Positive Predictive Value (PPV) | 0.00 | 0.19 | 0.35 | 0.48 | 0.59 | 0.68 | 0.76 | 0.83 | 0.89 | 0.95 | 1.00 |
|  | Negative Predictive Value (NPV) | 1.00 | 0.95 | 0.90 | 0.84 | 0.77 | 0.69 | 0.60 | 0.49 | 0.36 | 0.20 | 0.00 |
| GAD-7 (15-19 year olds), Cut-off ≥8, Sensitivity=0.70, Specificity=0.71 | Screening Tool Detected Prevalence | 0.29 | 0.33 | 0.37 | 0.41 | 0.45 | 0.50 | 0.54 | 0.58 | 0.62 | 0.66 | 0.70 |
|  | Difference between true prevalence and detected prevalence | 0.29 | 0.23 | 0.17 | 0.11 | 0.05 | -0.01 | -0.06 | -0.12 | -0.18 | -0.24 | -0.30 |
|  | Positive Predictive Value (PPV) | 0.00 | 0.21 | 0.38 | 0.51 | 0.62 | 0.71 | 0.78 | 0.85 | 0.91 | 0.96 | 1.00 |
|  | Negative Predictive Value (NPV) | 1.00 | 0.96 | 0.90 | 0.85 | 0.78 | 0.70 | 0.61 | 0.50 | 0.37 | 0.21 | 0.00 |
| PHQ-9  (12-14 year olds),  Cut-off ≥13 , Sensitivity=0.93, Specificity=0.80 | Screening Tool Detected Prevalence | 0.20 | 0.27 | 0.35 | 0.42 | 0.49 | 0.57 | 0.64 | 0.71 | 0.78 | 0.86 | 0.93 |
|  | Difference between true prevalence and detected prevalence | 0.20 | 0.17 | 0.15 | 0.12 | 0.09 | 0.06 | 0.04 | 0.01 | -0.02 | -0.04 | -0.07 |
|  | Positive Predictive Value (PPV) | 0.00 | 0.34 | 0.54 | 0.67 | 0.76 | 0.82 | 0.87 | 0.92 | 0.95 | 0.98 | 1.00 |
|  | Negative Predictive Value (NPV) | 1.00 | 0.99 | 0.98 | 0.96 | 0.94 | 0.92 | 0.88 | 0.83 | 0.74 | 0.56 | 0.00 |
| PHQ-9 (15-19 year olds),  Cut-off ≥11, Sensitivity=0.89, Specificity=0.70 | Screening Tool Detected Prevalence | 0.30 | 0.36 | 0.42 | 0.48 | 0.54 | 0.60 | 0.65 | 0.71 | 0.77 | 0.83 | 0.89 |
|  | Difference between true prevalence and detected prevalence | 0.30 | 0.26 | 0.22 | 0.18 | 0.14 | 0.10 | 0.05 | 0.01 | -0.03 | -0.07 | -0.11 |
|  | Positive Predictive Value (PPV) | 0.00 | 0.25 | 0.43 | 0.56 | 0.66 | 0.75 | 0.82 | 0.87 | 0.92 | 0.96 | 1.00 |
|  | Negative Predictive Value (NPV) | 1.00 | 0.98 | 0.96 | 0.94 | 0.91 | 0.86 | 0.81 | 0.73 | 0.61 | 0.41 | 0.00 |

Table 4 Discriminant ability of PHQ-A items for adolescents with and without diagnosis on K-SADS

| Item# Description | No diagnosis N=241 | Any Depression diagnosis  N= 51 |  |  |
| --- | --- | --- | --- | --- |
|  | Mean (SD) | Mean (SD) | t-test | p-value |
| ***Boys*** | **N= 129** | **N= 11** |  |  |
| PHQ 1 Feeling down, depressed, irritable, or hopeless | 0.69 (0.82) | 1.82 (0.87) | -4.37 | <.001 |
| PHQ 2 Little interest or pleasure in doing things | 0.58 (0.84) | 1.55 (0.93) | -3.60 | <.001 |
| PHQ 3 Trouble falling asleep, or staying asleep or sleeping too much | 0.53 (0.80) | 1.36 (0.92) | -3.29 | 0.001 |
| PHQ 4 Poor appetite, weight loss, or overeating | 0.43 (0.71) | 1.36 (0.81) | -4.19 | <.001 |
| PHQ 5 Feeling tired, or having little energy | 0.60 (0.84) | 1.91 (0.94) | -4.91 | <.001 |
| PHQ 6 Feeling bad about yourself /that you are a failure / letting people down | 0.60 (0.96) | 2.00 (0.78) | -4.67 | <.001 |
| PHQ 7 Trouble concentrating on things / school, work, reading | 0.76 (0.95) | 1.45 (0.93) | -2.33 | 0.021 |
| PHQ 8 Moving or speaking slowly / being fidgety or restless | 0.40 (0.82) | 1.27 (1.19) | -2.38 | 0.037 |
| PHQ 9 Thoughts that you would be better off dead / hurting yourself | 0.24 (0.57) | 1.10 (1.18) | -2.25 | 0.050 |
| ***Girls*** | **N= 112** | **N= 40** |  |  |
| PHQ 1 Feeling down, depressed, irritable, or hopeless | 0.93 (0.82) | 2.13 (0.76) | -8.04 | <.001 |
| PHQ 2 Little interest or pleasure in doing things | 0.80 (0.92) | 1.85 (0.77) | -6.44 | <.001 |
| PHQ 3 Trouble falling asleep, or staying asleep or sleeping too much | 0.63 (0.85) | 1.75 (0.93) | -6.97 | <.001 |
| PHQ 4 Poor appetite, weight loss, or overeating | 0.63 (0.88) | 2.05 (0.82) | -8.95 | <.001 |
| PHQ 5 Feeling tired, or having little energy | 0.84 (0.97) | 2.20 (0.79) | -7.95 | <.001 |
| PHQ 6 Feeling bad about yourself /that you are a failure / letting people down | 0.77 (0.93) | 2.17 (0.87) | -8.34 | <.001 |
| PHQ 7 Trouble concentrating on things / school, work, reading | 0.93 (0.97) | 2.28 (0.72) | -8.06 | <.001 |
| PHQ 8 Moving or speaking slowly / being fidgety or restless | 0.41 (0.75) | 1.55 (0.96) | -7.80 | <.001 |
| PHQ 9 Thoughts that you would be better off dead / hurting yourself | 0.44 (0.77) | 1.88 (1.11) | -7.50 | <.001 |

Table 5 Discriminant ability of GAD-7 items for adolescents with and without diagnosis on the K-SADS

| Item# Description | No diagnosis  N=241 | Any GAD diagnosis N= 102 |  |  |
| --- | --- | --- | --- | --- |
|  | Mean (SD) | Mean (SD) | t-test | p-value |
| ***Boys*** | **N= 129** | **N=28** |  |  |
| GAD 1 Feeling nervous. anxious or on edge | 0.60 (0.79) | 1.75 (0.80) | -6.98 | <0.001 |
| GAD 2 Not being able to stop or control worrying | 0.41 (0.85) | 1.25 (1.04) | -4.007 | <0.001 |
| GAD 3 Worrying too much about different things | 0.48 (0.85) | 1.46 (0.96) | -5.43 | <0.001 |
| GAD 4 Trouble relaxing | 0.36 (0.72) | 1.46 (1.07) | -5.19 | <0.001 |
| GAD 5 So restless that it is hard to sit still | 0.40 (0.81) | 1.25 (1.01) | -4.21 | <0.001 |
| GAD 6 Easily annoyed / irritable | 0.92 (1.01) | 1.79 (1.07) | -4.05 | <0.001 |
| GAD 7 Feeling afraid as if something awful might happen | 0.57 (0.94) | 0.89 (0.73) | -1.68 | 0.094 |
| ***Girls*** | **N= 112** | **N=74** |  |  |
| GAD 1 Feeling nervous. anxious or on edge | 0.88 (0.90) | 1.78 (0.78) | -7.04 | <0.001 |
| GAD 2 Not being able to stop or control worrying | 0.67 (0.91) | 1.65 (0.93) | -7.11 | <0.001 |
| GAD 3 Worrying too much about different things | 0.90 (1.03) | 1.84 (0.89) | -6.58 | <0.001 |
| GAD 4 Trouble relaxing | 0.62 (0.83) | 1.61 (0.93) | -7.59 | <0.001 |
| GAD 5 So restless that it is hard to sit still | 0.56 (0.88) | 1.45 (1.01) | -6.15 | <0.001 |
| GAD 6 Easily annoyed / irritable | 1.15 (1.08) | 1.95 (0.95) | -5.29 | <0.001 |
| GAD 7 Feeling afraid as if something awful might happen | 0.79 (0.94) | 1.57 (1.02) | -5.35 | <0.001 |
